# Traditional Chinese medicine-induced cardiac adverse reactions: a critical review of current evidence, knowledge gaps, and pharmacovigilance strategies

**DOI:** 10.3389/fphar.2026.1856615

**Published:** 2026-07-24

**Authors:** Xiaoxiao Cai, Kunchuwake Yasien, Ruoyu Gan, Qian Zhou, Bing Zhang, Yu Wang, Xiaomeng Zhang, Zhijian Lin

**Affiliations:** School of Chinese Materia Medica, Beijing University of Chinese Medicine, Beijing, China

**Keywords:** cardiac adverse reactions, pharmacovigilance, risk assessment, safe medication, toxic mechanisms, traditional Chinese medicine

## Abstract

Traditional Chinese Medicine (TCM) holds considerable promise for managing cardiovascular conditions, but its cardiac safety continues to raise legitimate clinical concerns. In this critical review, we synthesize what is currently known about TCM related cardiac adverse reactions, including epidemiological patterns, offending botanical drug metabolites, toxicological pathways, and pharmacovigilance practices. What emerges from our systematic evaluation is a body of evidence that is surprisingly thin in key areas, with knowledge gaps that seriously undermine both risk assessment and clinical decision making. We argue that the field relies too heavily on descriptive case reports and spontaneous notifications, lacks validated causality tools suited to complex botanical drug matrices, and has yet to translate mechanistic discoveries into actionable predictive models. Methodological weaknesses are pervasive: pharmacokinetic and pharmacodynamic interaction data are scarce, real world evidence remains underused in regulatory contexts, and no specific biomarkers exist for early detection of cardiac injury. Preclinical findings, moreover, are difficult to extrapolate to human physiology, and post marketing surveillance systems lag far behind what is needed. In response, we outline a practical agenda that prioritises TCM tailored pharmacovigilance protocols, leverages advanced analytical and computational platforms, and calls for stronger international data sharing frameworks. Our ultimate aim is not merely to catalogue problems, but to offer a critical yet constructive foundation for advancing the cardiac safety science of TCM, so that its integration into global healthcare can proceed on firmer, more evidence based ground.

## 1 Introduction

Traditional Chinese medicine (TCM), characterized by its multi-metabolite and multi-target nature, is extensively used in preventing and treating cardiovascular diseases, including arrhythmia, myocardial ischemia, and heart failure. However, its potential to induce adverse cardiac reactions, including cardiotoxicity, remains a critical concern. Cardiotoxicity manifests as arrhythmias, myocardial damage, and heart failure, and the complexity of TCM formulations poses challenges in fully elucidating cardiac safety profiles. Certain botanical drug metabolites, such as aconitine from *Aconitum carmichaelii* Debeaux, may exert both therapeutic and toxic effects, underscoring the balance between efficacy and safety (Wang D. et al., 2022; Zhou et al., 2021).

Advanced methodologies, including network pharmacology, molecular docking, and omics technologies, have been employed to dissect cardiotoxic potential. Network pharmacology has identified key pathways like PI3K/Akt and MAPK signaling in botanical drug-related cardiac effects (Wang F. et al., 2021; Jiao et al., 2024). Epigenetic modifications and oxidative stress pathways are also critical mediators (Zhou et al., 2025; Lv et al., 2022). Botanical drugs have shown promise in mitigating chemotherapy-induced cardiotoxicity (CIC) through antioxidation, anti-inflammation, and mitochondrial protection (Li et al., 2026; Shi et al., 2024; Jin et al., 2025), though the quality of evidence varies (Li C. et al., 2023; Liu et al., 2020).

**FIGURE 1:**
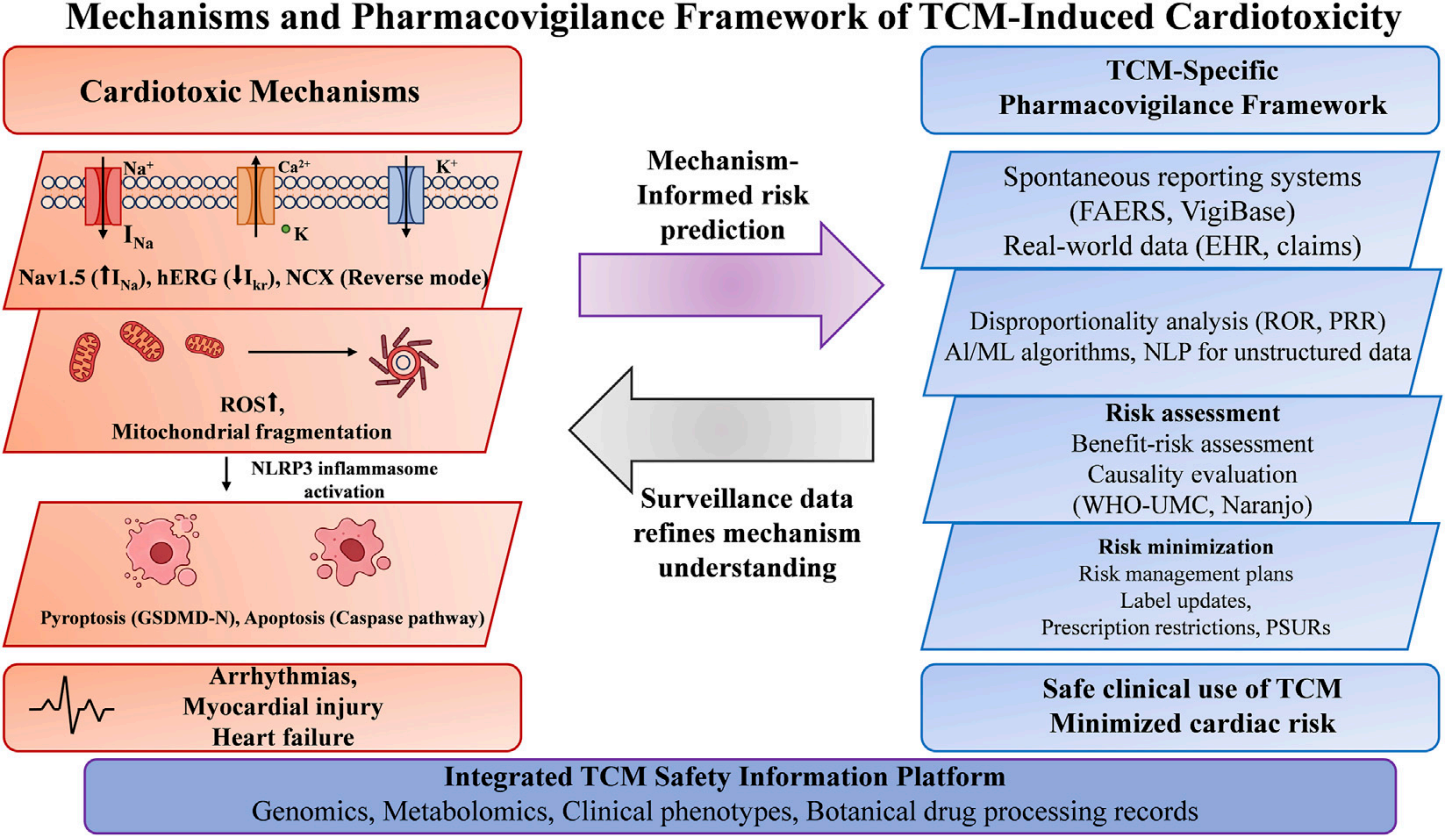
Synergistic framework of cardiotoxic mechanisms and pharmacovigilance.

Botanical drugs also modulate arrhythmias via effects on cardiac ion channels (Gao et al., 2025; Wang X. et al., 2021; Yao et al., 2020), but improper use may induce arrhythmias (Li et al., 2020a; Zhang WZ. et al., 2022). The lack of specific biomarkers complicates safety assessment, though microRNAs and metabolites show potential (Gu et al., 2025). Advances in vitro models like hiPSC-derived cardiomyocytes and organoids offer improved assessment platforms (Sun et al., 2023). Challenges remain in establishing robust pharmacovigilance systems, standardizing quality, and evaluating botanical drug-biomedicine interactions (Shen et al., 2024). This review provides a comprehensive overview of TCM-induced cardiac adverse reactions and proposes strategies for improved pharmacovigilance (Figure 1).

### 1.1 Literature search strategy and selection criteria

A comprehensive literature search was conducted in PubMed from database inception to April 2026 using MeSH terms and keywords: (Traditional Chinese Medicine OR botanical drug OR commercial Chinese polyherbal preparation) AND (cardiotoxicity OR arrhythmia OR QT prolongation OR pharmacovigilance). We included peer-reviewed studies detailing original clinical data, cardiac ion channel mechanisms, or validated botanical taxonomy, while excluding redundant or purely descriptive reports. From the initially retrieved records, 78 high-quality articles were selected for critical evaluation.

## 2 Epidemiological and current status analysis of adverse cardiac reactions induced by traditional Chinese medicine

### 2.1 Overview of domestic and international reports and data characteristics

Analysis of adverse drug reaction (ADR) reports from the China National Adverse Drug Reaction Monitoring Center and WHO VigiBase reveals that while the proportion of cardiotoxicity reports attributed to TCM is lower than for chemical drugs, the severity of some cases—hospitalization or life-threatening events—raises significant concern. Cardiac ADRs linked to Chinese materia medica (CMM) often co-occur with nervous and digestive system reactions (Zhang et al., 2020a). Reports predominantly originate from regions with high TCM utilization. Systemic challenges include high underreporting rates, variable report quality, and difficulty in causality assessment, which undermine epidemiological accuracy (Li C. et al., 2023). Comparative analyses show TCM accounts for a smaller fraction of total cardiotoxicity reports, yet the proportion of severe events within TCM-related cases is noteworthy. For instance, Shengmai injection (composed of *Panax ginseng* C.A.Mey. (Araliaceae), *Ophiopogon japonicus* (Thunb.) Ker Gawl. (Asparagaceae), and *Schisandra chinensis* (Turcz.) Baill. (Schisandraceae) demonstrates both therapeutic benefits and potential risks (Liu et al., 2020). Mechanistic studies have elucidated cardiotoxicity pathways involving hERG channel blockade and oxidative stress (Liang et al., 2025; Lu et al., 2025). Integrating real-world data with mechanistic studies is essential for enhanced understanding (Gu et al., 2025; Lv L. et al., 2023). Addressing underreporting and variable report quality through enhanced infrastructure and standardized protocols is critical for improving global safety monitoring of TCM-induced cardiac adverse reactions.

While ADR databases offer a useful starting point, their heavy dependence on passive reporting introduces unavoidable biases. Underreporting and incomplete records are well recognised problems that probably distort the true epidemiological picture of TCM induced cardiotoxicity. The absence of globally standardised reporting criteria for TCM specific adverse events further complicates cross regional comparisons and weakens the reliability of safety signals. As a result, the current evidence base cannot support definitive risk stratification. A deliberate move towards active surveillance and more refined analytical methods is needed to overcome these inherent limitations.

### 2.2 Common types of botanical drugs involved and their dosage forms

Botanical drugs implicated in cardiac adverse reactions include those containing cardiac glycosides (e.g., Chan Su), alkaloids (e.g., Wu Tou, *Aconitum carmichaelii* Debeaux (Ranunculaceae); Lei Gong Teng, *Tripterygium wilfordii* Hook. f. (Celastraceae)), and anthraquinone metabolites (e.g., Da Huang, encompassing *Rheum palmatum* L. (Polygonaceae), *Rheum officinale* Baill. (Polygonaceae), and *Rheum tanguticum* (Maxim. ex Regel) Balf. (Polygonaceae); He Shou Wu, *Polygonum multiflorum* Thunb. (Polygonaceae)) (Wang D. et al., 2022; Zhou et al., 2021). These botanical drugs are often used in metabolite formulations, which may metabolite risk due to synergistic toxicities. The risk varies significantly with dosage form. Injection preparations are particularly high-risk due to rapid systemic absorption, leading to swift and severe reactions; for example, Cinobufotalin injection has been linked to arrhythmias and myocardial injury (Li et al., 2020a). Oral formulations generally exhibit slower absorption but still pose risks with chronic or high-dose use (Wang D. et al., 2022). External preparations carry lower systemic risk, though absorption through damaged skin remains possible (Zhao Y. et al., 2024).

Clinical practices deviating from recommended usage significantly elevate risk. Off-label dosing, prolonged high-dose administration, and inconsistent botanical drug quality are critical contributing factors. The narrow therapeutic index of metabolites like aconitine underscores the dangers of overdose (Zhou et al., 2021). Variability in botanical drug quality due to cultivation, harvesting, and processing leads to unpredictable toxic metabolite concentrations (Wang D. et al., 2022). Rational clinical application with standardized quality control, dosage regulation, and vigilant pharmacovigilance is essential to minimize cardiotoxic risks (Wang D. et al., 2022; Li C. et al., 2023).

The identification of high risk botanical drug categories provides some guidance, but this knowledge rests largely on isolated case reports and has not been systematically validated at the population level. More importantly, the clinical relevance of formulation complexity and dosage form variability is often discussed without supporting pharmacokinetic data that could explain differential toxicity. This descriptive gap points to a clear research priority: controlled clinical studies that directly compare systemic exposure and cardiac effects across different dosage forms, moving beyond association to establish causation.

## 3 Potential material basis and chemical metabolites of cardiotoxicity induced by traditional Chinese medicine

### 3.1 Comprehensive classification and material basis of cardiotoxic metabolites

The intrinsic cardiotoxicity of TCMs is governed by a diverse spectrum of botanical matrices, comprising well-established toxic metabolites, trace metabolites, and dynamically transformed metabolites.

Classic toxic metabolites notably include specific alkaloid categories, such as aconitine-type diterpenoid alkaloids, indole alkaloids (e.g., triptolide), and pyrrolizidine alkaloids. The specific chemical configurations of these molecules dictate their binding affinity and pathologic interactions with cardiac ion channels and downstream cellular targets (Xiang et al., 2021; Shan et al., 2024). Beyond these prominent, well-documented toxins, recent toxicological investigations have unmasked latent or novel toxic metabolites buried within complex botanical drug matrices. These include minor hazardous metabolites found in botanical drugs like Radix Paeoniae Alba (from *Paeonia lactiflora* Pall [Paeoniaceae]) and Cortex Periplocae (from *Periploca sepium* Bunge [Apocynaceae]), as well as transient or stable toxic metabolites generated post-administration (Yang et al., 2021; Yu et al., 2024). Crucially, certain initially non-toxic parent metabolites present in raw botanical drugs can undergo structural modifications via hepatic or systemic metabolic pathways, yielding toxic metabolites that directly trigger delayed or idiosyncratic cardiotoxicity (Bai et al., 2022).

The presentation and threshold of these cardiotoxic metabolites are dynamically modulated by traditional processing (paozhi) and multi-botanical drug compatibility. Hydrothermal processing methods, including boiling or steaming, induce the cleavage of ester bonds in highly toxic diester diterpene alkaloids (DDAs), converting them into less toxic monoester or non-esterified derivatives (e.g., transforming aconitine into benzoylaconine or aconine), thereby preserving therapeutic merit while mitigating acute lethal toxicity (Shan et al., 2024; Yan et al., 2020; Lei et al., 2024; He et al., 2022). Nevertheless, trace amounts of residual DDAs can still escape degradation, posing clinical risks of cumulative cardiotoxicity during long-term or high-dose therapy (Lu et al., 2022).

Furthermore, multi-metabolite multi-botanical drug interactions within TCM formulae play a dual role in toxicity modulation. Synergistic interactions among co-existing metabolites can aggravate cardiac risks, whereas antagonistic combinations serve as a detoxification mechanism. For instance, the co-formulation of Fuzi (*Aconitum carmichaelii* Debeaux [Ranunculaceae]) with Glycyrrhiza (*Glycyrrhiza uralensis* Fisch. ex DC [Fabaceae]) decreases the free concentration of toxic aconitineand exerts concomitant anti-inflammatory protection, demonstrating compatibility-driven detoxification, while specific botanical drug-derived flavonoids can stabilize ion channels to counteract alkaloid-induced disruption (Yan et al., 2020; Lei et al., 2024; Jiang et al., 2022). Additionally, minor or trace metabolites may transition from dormant to active toxic states under specific clinical contexts, such as individual microecological variability (e.g., gut microbiota shifts) or environmental fluctuations, which alter the bioavailability and target susceptibility of these metabolites (He et al., 2020).

The characterisation of toxic metabolites lays a necessary chemical groundwork, yet the field tends to overemphasise individual metabolites while neglecting their contextual behaviour within the complex matrix of a full botanical decoction. Translating *in vitro* and *in vivo* findings into clinically meaningful risk predictions remains difficult, largely because the absorption, distribution, metabolism and excretion profiles of these metabolites in humans are poorly understood. In addition, the potential influence of the exposome, including dietary habits and environmental exposures, constitutes a largely unexplored domain that may significantly modulate cardiotoxic outcomes.

### 3.2 Advanced methodologies and analytical technologies for cardiotoxic metabolite identification

Accurately decoding the intricate and trace material basis of TCM-induced cardiotoxicity requires highly sensitive, high-throughput, and dynamic analytical platforms capable of isolating and characterizing hazardous agents from complex multi-metabolite backgrounds.

Modern analytical frameworks, anchored by high-resolution mass spectrometry (HRMS) and nuclear magnetic resonance (NMR), have revolutionized the structural elucidation and discovery of both prominent and trace cardiotoxins (Yang et al., 2021; Yu et al., 2024; Bai et al., 2022). For rapid profiling and untargeted screening, Ultra-High-Performance Liquid Chromatography coupled with Quadrupole Time-of-Flight Mass Spectrometry (UHPLC-Q-TOF-MS) provides an essential platform. This approach enables high-throughput identification and chemical classification of potentially cardiotoxic metabolites within multi-metabolite botanical drug extracts prior to clinical use (Yang et al., 2021).

To capture and characterize highly unstable, transient, or reactive intermediates—which are frequently implicated in idiosyncratic cardiac injury—liquid chromatography-tandem mass spectrometry (LC-MS/MS) paired with chemical trapping strategies represents the gold standard. For instance, utilizing glutathione (GSH) trapping techniques allows for the stable conjugation and subsequent mass-spectrometric tracing of reactive metabolites derived from complex botanical drug structures like cardiac glycosides (Yu et al., 2024).

Furthermore, mass spectrometry-driven metabolomics has emerged as a cornerstone strategy for systemic pharmacovigilance. It enables researchers to map the dynamic *in vivo* metabolic fates of TCM metabolites, delineate precise biotransformation pathways, and distinguish downstream cardiotoxic metabolites from their non-toxic precursor metabolites (Bai et al., 2022). Looking forward, an integrated analytical paradigm that seamlessly bridges HRMS structural screening, dynamic metabolomics tracking, and host microecological assessments constitutes the ultimate methodological framework to comprehensively identify and monitor clinically significant toxic metabolites in TCM (He et al., 2020).

## 4 Research on the mechanisms and pathophysiology of cardiotoxicity induced by traditional Chinese medicine

### 4.1 Mechanisms at the cellular and molecular levels

Toxic botanical drug metabolites exert direct effects on myocardial ion channels, disrupting cardiac electrophysiology and leading to arrhythmias. Alkaloids from Fritillaria species (e.g., *Fritillaria cirrhosa* D. Don [Liliaceae] and *Fritillaria thunbergii* Miq [Liliaceae]) block hERG potassium channels, prolonging repolarization and increasing ventricular arrhythmia risk (Lu et al., 2025). Aconitine induces ventricular arrhythmias by activating Notch1 signaling, which epigenetically upregulates HCN4 expression in cardiomyocytes, disrupting pacemaker activity (Zhou et al., 2020). Metabolites from various botanical drugs also show binding affinities to cardiac calcium channels (Yang et al., 2025). These findings underscore that toxic metabolites directly interfere with myocardial ion channels, causing action potential alterations and conduction abnormalities.

Beyond ion channel modulation, toxic metabolites induce cardiotoxicity through intracellular stress pathways. Oxidative stress, characterized by excessive ROS generation, damages cellular macromolecules and organelles. Periplocin and bufalin impair mitochondrial function and energy metabolism by dysregulating the AMPK/SIRT1/PGC-1α axis, leading to excessive autophagy and cardiomyocyte death (Gao et al., 2026). Key apoptotic regulators like the caspase family, Bcl-2/Bax ratio, and MAPK pathways are modulated by botanical drug metabolites (Xu et al., 2021; Zhai et al., 2024). Interference with the cardiac autonomic nervous system further contributes to heart rate and rhythm abnormalities, although direct evidence remains limited (Zhou et al., 2020; Sun et al., 2021). Elucidating these multifaceted mechanisms provides a foundation for targeted safety evaluations and pharmacovigilance strategies.

Aconitine and mesaconitine bind to Nav1.5 channels, accelerating activation and preventing inactivation, thereby inducing a pathological late sodium current; simultaneously, they inhibit Na^+^/K^+^-ATPase activity, leading to intracellular Na^+^ accumulation. Elevated Na^+^ reverses the sodium-calcium exchanger, causing cytosolic Ca^2+^ overload and delayed afterdepolarizations, which on ECG manifest as premature ventricular contractions, atrioventricular block, ventricular tachycardia, and fibrillation (Jiang et al., 2022; Wang XC. et al., 2021). During repolarization, the hERG potassium channel is a key target: isosteroidal alkaloids from *Fritillaria* species (peimine, peiminine, sipeimine) physically block the hERG channel pore, depleting repolarization reserve and prolonging action potential duration, clinically presenting as QT prolongation and predisposing to early afterdepolarizations and torsade de pointes (TdP) (Lu et al., 2025). In addition, bufalin concentration-dependently enhances the late sodium current (I_NaL) and increases Na^+^-Ca^2+^ exchange current, contributing to its arrhythmogenic effects (Li et al., 2020b). These multi-channel effects are summarized in Table 1.

**TABLE 1:** Cardiotoxic metabolites of TCM, primary ion channel targets, electrophysiological effects, and associated arrhythmia types.

Botanical drug metabolite	Source	Primary targets	Effect on currents	Induced arrhythmias
Aconitine	*Aconitum carmichaelii* Debeaux (Ranunculaceae) (Fuzi, Chuanwu)	Nav1.5 (↑I_Na) Na^+^/K^+^-ATPase (↓I_Na/K)	Accelerates sodium channel activation; intracellular Na^+^ accumulation, Ca^2+^ overload, DADs	Premature ventricular contractions, conduction block, VT, VF
Mesaconitine	*Aconitum carmichaelii* Debeaux (Ranunculaceae) (Fuzi, Chuanwu)	Nav1.5 (↑I_Na) Na^+^/K^+^-ATPase (↓I_Na/K)	Accelerates sodium channel activation (more potent than aconitine); intracellular Na^+^ accumulation	Premature ventricular contractions, conduction block, VT, VF
Peimine, peiminine, sipeimine	*Fritillaria* species (liliaceae) (chuanbeimu, zhebeimu)	hERG (I_Kr, ↓)	Physical block of hERG pore; IC50 values: 26.1 μM, 36.8 μM, 47.6 μM; antagonistic interactions among the three	QT prolongation, EADs, torsade de pointes (TdP)
Bufalin	Chan su (toad venom)	Nav1.5 (↑I_NaL) Na^+^/K^+^-ATPase (↓)	Concentration-dependent enhancement of late sodium current; increased Na^+^/Ca^2+^ exchange	Torsade de pointes, VT

Mechanistic studies have advanced our understanding of molecular pathways, but the prevailing reliance on reductionist models tends to overlook how multi-metabolite interactions may produce synergistic or antagonistic effects at the whole-heart level. Ion channel assays and cell-based stress models, while valuable, frequently fail to predict the integrated cardiac responses observed *in vivo*, such as complex arrhythmias. Future work should strive to bridge this gap by correlating molecular findings with tissue-level and organ-level functional assessments, thereby building a more comprehensive and predictive safety framework.

### 4.2 Pathophysiological effects at the organ and system levels

Acute toxicity induced by certain botanical drug metabolites can precipitate severe cardiac events such as acute heart failure or cardiogenic shock through myocardial contractility suppression and coronary artery spasm. Podophyllotoxin (PPT) causes acute cardiotoxicity characterized by rapid onset of cardiac injury marker elevation and histopathological alterations, involving oxidative stress, apoptosis, and inflammation (Ma et al., 2024; Liu C. et al., 2025).

Chronic or cumulative exposure may lead to progressive structural cardiac remodeling, including myocardial fibrosis and hypertrophy. Long-term administration of Arecae semen aqueous extract has been associated with metabolic disturbances contributing to chronic cardiotoxicity (Jia et al., 2020).

Indirectly, toxic botanical drug metabolites may impair cardiac function through systemic inflammatory responses, electrolyte imbalances, and hepatic or renal dysfunction. Podophyllotoxin-induced cardiotoxicity involves gut microbiota remodeling and metabolic profile alterations (Sun L. et al., 2025). Systemic inflammation elevates pro-inflammatory cytokines like TNF-α and IL-6, promoting adverse cardiac remodeling (Zhang et al., 2024). Electrolyte disturbances and organ impairment further compromise cardiac electrophysiological stability. These indirect pathways underscore the complexity of TCM-induced cardiotoxicity, where multi-organ interactions contribute significantly to adverse outcomes.

Descriptions of organ-level pathophysiology tend to be largely descriptive and do not always establish a clear, linear relationship between the molecular events identified and the clinical phenotypes observed in patients. Causality is often inferred from temporal association, leaving substantial uncertainty about the time-course and the critical junctures that link subcellular injury to overt organ dysfunction. A systems-level integration of mechanistic data is urgently needed to connect these discrete observations and to pinpoint clinically actionable targets for intervention.

## 5 Risk factors and susceptible populations for adverse cardiac reactions to traditional Chinese medicine

### 5.1 Botanical drug-related factors

The quality and safety of botanical drug materials are influenced by botanical source, cultivation, harvesting, adulteration, and processing methods. Confusion of botanical origins, such as misidentification of *Aconitum* species with varying alkaloid concentrations, can increase cardiotoxic risks (Liang et al., 2025). Environmental pollution with heavy metals and pesticides, improper harvest timing, and illegal addition of biomedical pharmaceuticals further compromise safety (Wang D. et al., 2022).

Pharmaceutical processing significantly affects toxic metabolite content. Processing of Fuzi reduces toxic diester diterpene alkaloid levels through hydrolysis, attenuating cardiotoxicity while preserving efficacy (Lei et al., 2024). Variations in decoction time, temperature, and extraction methods influence the concentration of both toxic and active metabolites. Standardization of processing parameters is essential for consistent quality and safety (Wang X. et al., 2022).

Concomitant use of botanical drugs with conventional biomedicine introduces complex pharmacokinetic and pharmacodynamic interactions that can exacerbate cardiotoxicity. Co-administration of botanical drugs containing cardiac glycosides with digoxin increases digitalis toxicity risk due to additive effects on cardiac conduction (Wei et al., 2021). Altered drug metabolism via CYP450 enzymes and P-glycoprotein can lead to increased plasma concentrations of cardiotoxic metabolites (Lin et al., 2025). Rational evaluation of botanical drug-biomedicine interactions is critical to prevent adverse cardiac outcomes.

The identification of drug-related risk factors is an essential step, but the current knowledge remains largely qualitative and lacks the quantitative risk models needed to inform clinical decisions. Although processing methods and botanical drug-botanical drug interactions are known to modulate toxicity, the specific conditions under which they reliably reduce risk are not well defined, making standardisation difficult. More rigorous quantitative investigations and systematic reviews are required to transform these descriptive observations into evidence-based, actionable guidelines.

### 5.2 Patient-related factors

Individual patient-related factors critically modulate the risk and severity of TCM-induced adverse cardiac reactions. Genetic polymorphisms affecting drug metabolism enzymes (CYP450), drug transporters (P-glycoprotein), and ion channel-related genes are key determinants of inter-individual variability. Variants may lead to altered metabolic capacity, increased systemic exposure, or predisposition to arrhythmogenic effects (Ding et al., 2022).

Physiological and pathological conditions such as age, comorbidities, and electrolyte imbalances significantly impact cardiac tolerance. Elderly patients with diminished cardiac reserve and altered pharmacokinetics are more vulnerable. Pediatric populations may have immature metabolic pathways. Underlying cardiac, hepatic, or renal dysfunction can exacerbate cardiotoxic potential by impairing drug clearance. Electrolyte disturbances like hypokalemia and hypomagnesemia lower the threshold for arrhythmias (Fan et al., 2021).

Special physiological states such as pregnancy and lactation introduce additional complexities. Physiological changes during pregnancy can modify pharmacokinetics and pharmacodynamics, potentially enhancing cardiotoxic risks. Lactating women may transfer botanical drug metabolites through breast milk to neonates. Allergic predispositions may trigger exaggerated immune responses (Zhang et al., 2021). Understanding these factors is essential for individualized risk assessment and developing targeted pharmacovigilance strategies to minimize cardiotoxicity.

The importance of patient-related factors is widely acknowledged, yet our understanding is based predominantly on statistical associations rather than validated, predictive biomarkers. Genetic predispositions and comorbid conditions are frequently cited, but their clinical utility remains limited without practical diagnostic tools or algorithms for individualised risk assessment. Converting these broad epidemiological associations into a functional, personalised risk prediction model is a critical unmet need for the safe clinical application of TCM.

## 6 Clinical manifestations and diagnostic challenges of adverse cardiac reactions to traditional Chinese medicine

### 6.1 Clinical features and classification

The clinical spectrum of TCM-induced cardiac adverse reactions ranges from subtle, asymptomatic electrocardiographic abnormalities (QT prolongation, ST-T changes) to severe, life-threatening events. Symptomatic arrhythmias may manifest as palpitations, dizziness, or syncope. Severe presentations include myocardial injury with chest pain and elevated enzymes, heart failure with dyspnea and edema, and sudden cardiac death from malignant arrhythmias (Liu Y. et al., 2025).

Temporally, reactions are categorized as acute toxic responses or chronic cumulative injuries. Acute toxicity, such as that from aconitine alkaloids or toad toxin, often manifests rapidly with malignant arrhythmias and potential cardiac arrest (Liu Y. et al., 2025). Chronic cardiotoxicity develops insidiously over prolonged exposure, exemplified by cardiomyopathy from *Polygonum multiflorum* Thunb. (He Shou Wu, Polygonaceae), characterized by progressive myocardial fibrosis and eventual heart failure (Xiang et al., 2021).

A significant challenge is the often covert nature of early manifestations. Nonspecific symptoms like fatigue or mild chest discomfort can be easily overlooked. Experimental studies show that subclinical injury, involving oxidative stress and apoptosis, can progress rapidly to overt dysfunction (Ma et al., 2024). The complexity of TCM formulations, where cardioprotective flavonoid metabolites may mask cardiotoxic effects, further complicates identification and classification (Shi et al., 2024). Heightened clinical awareness and systematic monitoring are essential for early detection and risk stratification.

The clinical features of cardiotoxicity have been described in considerable detail, but the field still lacks a standardised, TCM-specific classification system that integrates both clinical presentation and underlying toxicological mechanisms. This absence of a unified framework complicates communication among clinicians and impedes the development of targeted diagnostic and therapeutic strategies. Early subclinical changes are particularly prone to being overlooked, and the nonspecific nature of many symptoms contributes to underdiagnosis. The development of validated, sensitive diagnostic tools should therefore be a high priority.

### 6.2 Diagnostic methods and challenges in causality assessment

Diagnosis of TCM-induced cardiac adverse reactions requires a multifaceted approach integrating detailed medication history, ECG dynamic monitoring, echocardiography, and biomarkers of myocardial injury (troponins, BNP). Blood drug concentration measurements, when feasible, can assist in correlating exposure with toxic effects.

Causality assessment presents unique challenges. Standardized tools like the WHO-UMC system and Naranjo Scale are complicated by the multi-metabolite nature of TCM formulations, making it difficult to attribute toxicity to a single metabolite. The absence of specific antidotes limits dechallenge and rechallenge testing. Individual variability in metabolism and genetic predisposition further complicates assessment, reducing the sensitivity and specificity of standard tools for TCM-related events.

Exclusion of alternative etiologies is critical. Primary cardiac diseases, concurrent use of other cardiotoxic medications, and infectious causes like viral myocarditis must be carefully ruled out through thorough differential diagnosis. Emerging diagnostic technologies, such as electrochemical immunosensors targeting HFABP and zebrafish models for toxicity screening, may enhance early detection and evaluation (Sun B. et al., 2025; Zhang Y. et al., 2022). Future efforts should focus on refining diagnostic criteria specific to TCM-induced cardiotoxicity, developing specific biomarkers, and enhancing pharmacovigilance systems.

## 7 Current traditional Chinese medicine safety monitoring systems and their limitations

### 7.1 National adverse drug reaction monitoring system

China’s national ADR monitoring system is a multi-tiered framework centered around the National Center for Adverse Drug Reaction Monitoring. This hierarchical, interconnected network enables data collection from grassroots facilities up to the national level. The system has contributed to regulatory actions, such as label revisions and withdrawals of high-risk TCM injections linked to cardiovascular events (Sun et al., 2023; Yao et al., 2025). However, inherent limitations of passive surveillance exist. Underreporting remains a significant challenge due to lack of awareness or time constraints among providers. Report quality and completeness are often suboptimal, frequently missing critical details about botanical drug source, processing, and compatibility. This hampers accurate causality assessment and risk stratification. Furthermore, limited capacity for advanced data mining restricts the prompt identification of subtle or emerging safety signals, delaying early warning capabilities (Zhuang et al., 2023). Optimizing operational efficiency and data robustness is essential to fully realize the system’s potential in protecting cardiovascular safety in TCM clinical practice.

Although a national pharmacovigilance system is in place, its effectiveness is seriously constrained by its passive reporting structure and the substantial underrecording of TCM related adverse events. The current system is not well equipped to capture the subtle, chronic or idiosyncratic cardiac effects that characterise many TCM formulations, rendering it a rather blunt instrument for safety monitoring. Fundamental reform is necessary to move towards a proactive, data driven surveillance model that makes full use of modern analytical capabilities and integrates real world clinical information.

### 7.2 Post-marketing studies and non-clinical re-evaluation

Post-marketing safety surveillance, including phase IV trials, focused monitoring, and real-world evidence studies, is critical for identifying cardiac adverse reactions not fully elucidated pre-approval. These modalities reveal rare or delayed events in larger, more diverse populations under routine conditions (Zhang et al., 2020b). Focused monitoring facilitates real-time tracking of specific cardiac safety signals. Real-world studies provide valuable data on incidence and risk factors in routine practice, complementing controlled trial data.

Non-clinical re-evaluation of older TCMs based on newly discovered toxicological mechanisms is increasingly necessary. Supplementary cardiac safety pharmacology studies using *in vitro* and *in vivo* models are employed to elucidate cardiotoxic potential. For example, water-grinding techniques for realgar have been shown to reduce toxicity, highlighting the importance of integrating modern toxicological assessments with traditional knowledge (Wang D. et al., 2022; Han et al., 2021).

Challenges hindering post-marketing research effectiveness include insufficient resource allocation, suboptimal study designs (inadequate sample sizes, lack of controls), and slow translation of findings into regulatory actions. The complexity of TCM formulations further complicates study design and interpretation. Addressing these issues requires enhanced investment in pharmacovigilance infrastructure, rigorous standardized methodologies, and streamlined regulatory pathways to ensure timely incorporation of new safety data into practice.

## 8 Core strategies and key technologies for pharmacovigilance of traditional Chinese medicine

### 8.1 Risk-based lifecycle management

Risk-based lifecycle management integrates pharmacovigilance principles throughout the entire development and post-marketing phases of TCM. This begins with rigorous preclinical toxicity screening to identify potential cardiotoxic metabolites in botanical drug formulations, such as steroidal saponins in Dioscorea bulbifera L. (Dioscoreaceae) (Mathew et al., 2026). During registration, benefit-risk assessments must meticulously balance therapeutic efficacy against potential cardiac adverse effects (Jin et al., 2025). Post-marketing surveillance monitors real-world safety data to identify new or rare cardiotoxic signals, extending through the product lifecycle.

To operationalize this for TCM, risk minimization action plans are essential. These should include detailed medication guidelines for clinicians and patients, educational materials to enhance awareness, and implementation of prescription qualification management or registries for high-risk products, especially TCM injections containing known toxic metabolites (Wang et al., 2024). Active surveillance mechanisms, including proactive monitoring and mandatory periodic safety update reports (PSURs), are crucial for ongoing safety evaluation. Recent comprehensive pharmacovigilance guidelines in China tailored to various TCM dosage forms incorporate international best practices, facilitating systematic signal detection, risk assessment, and control (Wang et al., 2024; Zhao XX. et al., 2024). These integrated measures collectively optimize the benefit-risk balance of TCM and safeguard public health.

The concept of risk-based lifecycle management is logically sound, yet its practical implementation for TCM encounters considerable obstacles, especially concerning the feasibility and standardisation of risk minimisation action plans across such a diverse range of formulations. The evidence supporting many of these strategies remains preliminary and is largely derived from conventional drug models, which may not be directly applicable to the unique properties of TCM. A dedicated effort to develop and validate TCM-specific risk management tools and regulatory science is essential if this framework is to move beyond theoretical appeal and become a practical reality.

### 8.2 Application of modern information technology and data analysis

The integration of modern information technology has revolutionized pharmacovigilance for TCM. Natural language processing (NLP) enables automatic extraction of suspected cardiac adverse reaction signals from unstructured data sources like electronic medical records and internet platforms. NLP algorithms can detect subtle patterns indicative of cardiotoxicity, enhancing signal detection efficiency and broadening surveillance scope.

Data mining and AI algorithms, including disproportionality analysis methods (ROR, PRR), facilitate the identification of potential risk signals from large-scale databases like FAERS and VigiBase. Such analyses have detected cardiotoxicity signals related to botanical drugs like matrine and evodiamine (Fang et al., 2024). Network meta-analyses integrating clinical trial data with pharmacovigilance databases provide comprehensive risk assessments (Wang HK. et al., 2022). AI-driven knowledge graphs and machine learning models enhance interpretability by contextualizing signals within biological pathways and clinical phenotypes.

The conceptualization of an integrated “TCM Safety Information Platform” represents a promising Frontier in precision pharmacovigilance. By amalgamating medication usage records, genomic information, and clinical phenotypes, such a platform would enable a holistic, personalized approach to cardiac safety monitoring. Incorporating pharmacogenomic data could identify genetic susceptibilities, facilitating risk stratification and individualized therapy adjustments (Liang et al., 2021). These technologies collectively promise to advance the accurate identification of risk signals and support personalized risk management strategies.

### 8.3 Laboratory testing and biomarker research

Advancements in laboratory testing and biomarker research are pivotal for enhancing detection and monitoring of TCM-induced cardiotoxicity. Developing rapid and sensitive assays for measuring blood concentrations of common toxic botanical drug metabolites (e.g., from Momordica cochinchinensis (Lour.) Spreng [Cucurbitaceae] or Fuzi [*Aconitum carmichaelii* Debeaux, Ranunculaceae]) can facilitate timely diagnosis and guide therapeutic interventions (Du et al., 2021; Yang et al., 2020).

Research into early and specific cardiac toxicity biomarkers is gaining momentum. High-sensitivity cardiac troponin T (hs-cTnT) shows promise for early diagnosis of cancer treatment-related cardiac dysfunction, though optimal cut-off values are still being established (Lv X. et al., 2023). Beyond conventional protein biomarkers, noncoding RNAs (e.g., miRNA-21–3p) and endogenous metabolites are emerging as sensitive and specific indicators of TCM-related cardiac injury, offering insights into mechanistic pathways and early-warning capabilities (Gu et al., 2025).

Novel *in vitro* models such as organoids and organ-on-a-chip systems represent a transformative approach to predicting cardiotoxicity with higher precision. While zebrafish models are used for high-throughput screening due to low cost and genetic similarity, physiological differences limit translational applicability (Zhang Y. et al., 2022). Human-relevant models like cardiac organoids and heart-on-a-chip recapitulate human tissue architecture and function, providing improved platforms for mechanistic studies and safer drug development (Ma et al., 2024). Future research should prioritize standardizing biomarker assays, validating novel biomarkers in clinical cohorts, and developing human-relevant platforms to bridge preclinical findings with clinical outcomes.

## 9 Future prospects for constructing a traditional Chinese medicine-specific pharmacovigilance system

### 9.1 Multidisciplinary collaboration and international integration

The complexity of TCM-induced cardiac adverse reactions necessitates robust multidisciplinary collaboration involving experts from pharmacy, medicine, toxicology, informatics, and epidemiology. This collaborative framework ensures comprehensive monitoring and evaluation, enabling timely risk identification and mitigation. Integration of these disciplines fosters innovation in methodologies like big data analytics and AI, enhancing adverse event detection sensitivity and specificity.

International collaboration with global regulatory agencies (EMA, FDA, WHO) is equally critical. Aligning TCM pharmacovigilance practices with international standards enhances data comparability and interoperability, facilitating cross-border sharing of adverse reaction reports and safety signals. This global data exchange accelerates the identification of rare or delayed cardiotoxic effects. Harmonizing terminology and causality assessment methods promotes mutual recognition of safety data, supporting worldwide regulatory decision-making.

To sustain these efforts, incorporating pharmacovigilance concepts into the education and continuing professional development of TCM practitioners and researchers is imperative. Embedding training within higher education curricula and continuing education programs cultivates a cadre of specialized personnel capable of bridging traditional knowledge with modern safety science, enhancing the overall quality and reliability of TCM pharmacovigilance systems (Chen et al., 2025; Miao et al., 2025).

### 9.2 Strengthening basic research and translational application

The complexity of TCM systems necessitates intensified foundational research to elucidate toxic metabolites, mechanisms of action, and individual susceptibility factors. Advanced models like zebrafish are useful for high-throughput screening, but precise toxicological characterization of specific metabolites like aconitine is crucial (Zhou et al., 2021; Zhang Y. et al., 2022). Identifying molecular targets and pathways involved in oxidative stress, inflammation, apoptosis, and epigenetic modifications has been pivotal for guiding safer clinical applications (Zhou et al., 2025; Lv et al., 2022). Network pharmacology and molecular docking approaches have mapped metabolite-target interactions, revealing core targets and key metabolites instrumental in mediating cardiotoxic or cardioprotective effects (Dan et al., 2020).

Translational application is critical to bridge the gap between laboratory discoveries and clinical or regulatory practice. Developing cardiotoxicity prediction models specific to TCM, integrating multi-omics data and computational toxicology, represents a promising direction. AI tools combined with molecular docking can predict cardiotoxicity of botanical drug metabolites, enabling rapid identification of potentially harmful metabolites (Yang et al., 2025). Creating rapid detection kits for cardiotoxic metabolites can facilitate real-time monitoring and early warning in clinical settings (Li C. et al., 2023). A novel and culturally congruent approach involves leveraging TCM theory of “syndrome (zheng)-constitution” combined with modern biological markers to establish personalized risk assessment models (Gu et al., 2025; Liu et al., 2024). This integrative strategy could enable tailored therapeutic regimens, minimizing adverse cardiac events while preserving the holistic benefits of TCM.

### 9.3 Improving regulatory policies and social co-governance

Enhancing regulatory frameworks and promoting social co-governance are critical for ensuring TCM safety. A fundamental step involves revising technical guidelines for TCM safety evaluation to include explicit requirements for cardiac safety studies. Current pharmacovigilance guidelines for commercial Chinese polyherbal preparations (CCPPs) emphasize the need for systematic monitoring, signal detection, and risk control adapted to TCM characteristics (Liu et al., 2024; Fan et al., 2024; Fu et al., 2024). Clearly defining cardiac safety evaluation criteria will ensure that preclinical and clinical studies adequately assess potential cardiotoxic effects.

Establishing a robust legal framework that delineates responsibilities and liabilities for adverse reaction reporting is essential. Implementing incentive mechanisms alongside legal obligations—such as recognition programs and streamlined reporting processes—can enhance reporting enthusiasm and compliance, thereby strengthening the pharmacovigilance database (Fan et al., 2024; Zheng et al., 2025).

Public education plays a pivotal role in fostering a social environment conducive to safe TCM use. Educational campaigns should focus on rational drug use, emphasizing dosage adherence, contraindications, and early signs of cardiotoxicity. Elevating public understanding of TCM’s benefits and risks cultivates a culture of shared responsibility and vigilance, promoting social co-governance in medication safety (Lv et al., 2022). Integrating real-world data and evidence-based evaluations into regulatory decision-making, as demonstrated by comprehensive clinical evaluations of formulations like Reyanning Mixture, can support continuous safety profile improvement (Lyu et al., 2022). These multifaceted strategies collectively contribute to a safer therapeutic environment for TCM users and mitigate the risk of cardiotoxicity.

## 10 Conclusion

This review has surveyed the current landscape of TCM induced cardiac adverse reactions, from epidemiology and toxic metabolites to mechanisms and pharmacovigilance practices. What emerges clearly from the literature is a field that has made considerable descriptive progress yet remains surprisingly immature in its capacity to predict, detect, and prevent cardiac harm in clinical practice. The evidence base, while substantial in volume, is disproportionately built upon case reports and spontaneous notifications, with rigorous quantitative studies and validated predictive tools conspicuously absent. Mechanistic insights into ion channel disruption, oxidative stress, and mitochondrial dysfunction have advanced our understanding considerably, but these findings have not been systematically translated into clinical biomarkers, risk stratification algorithms, or regulatory decision making frameworks. The pharmacovigilance infrastructure, despite recent regulatory efforts, continues to operate on a passive reporting model ill suited to capture the subtle, chronic, or idiosyncratic cardiac effects that characterise many TCM formulations.

Addressing these shortcomings requires a deliberate research agenda that moves beyond description towards prediction and prevention. We propose six priority areas that should guide future investigations. First, the development and validation of TCM specific causality assessment tools represents a foundational need, as current instruments derived from conventional drug models are poorly adapted to the complexities of multi-botanical drug formulations, leading to inconsistent and often unreliable attributions of adverse events. Second, the identification and clinical qualification of sensitive biomarkers for early cardiac injury, particularly those that can distinguish transient electrophysiological disturbances from irreversible myocardial damage, are urgently needed to enable timely intervention and to provide objective endpoints for safety studies. Third, systematic pharmacokinetic and pharmacodynamic interaction research must be prioritised, given that the majority of TCMs are used concomitantly with conventional biomedicine, yet the interaction profiles of even widely used botanical drugs remain largely uncharacterised. Fourth, the application of advanced analytical platforms, including high resolution mass spectrometry based metabolomics and AI driven computational toxicology, should be expanded to enable prospective cardiotoxicity screening of botanical drug metabolites and formulations before they reach the market. Fifth, post-marketing surveillance systems require fundamental reform, shifting from passive spontaneous reporting to active, data-driven monitoring that leverages electronic health records, registry data, and real-world evidence to capture the full spectrum of cardiac events. Sixth, international collaboration and data sharing frameworks must be strengthened to enable cross-regional safety signal detection, particularly for rare or delayed adverse effects that may not emerge within a single country’s surveillance system.

These six priorities are not independent but mutually reinforcing. Better biomarkers, for instance, would enhance the value of real-world data, while improved causality assessment would facilitate signal validation across international databases. Crucially, translating these foundational scientific discoveries into clinical practice will require parallel evolution in regulatory frameworks. Guidelines for quality control, standardisation, and post-marketing surveillance must be refined to accommodate the unique challenges posed by TCM formulations, ensuring that regulatory science keeps pace with mechanistic and analytical advances. The overarching goal is to move the cardiac safety science of TCM from a reactive, descriptive discipline towards a predictive, evidence based framework that can support confident clinical decision making. Achieving this vision will demand sustained commitment from researchers, clinicians, regulators, and funding bodies, as well as a willingness to adopt methodologies and standards from adjacent fields of biomedical science without losing sight of the distinctive characteristics of TCM. The challenges are substantial, but they are not insurmountable. Through integrated endeavours that harmonise research perspectives with clinical insights, it is possible to realise the therapeutic promise of TCM while ensuring that its cardiac risks are understood, communicated, and managed with the same rigour expected of any other metabolite of modern healthcare.
